# Self-Powered Photonic Synapses with Rapid Optical Erasing Ability for Neuromorphic Visual Perception

**DOI:** 10.34133/research.0526

**Published:** 2024-11-07

**Authors:** Mingchao Li, Chen Li, Kang Ye, Yunzhe Xu, Weichen Song, Cihui Liu, Fangjian Xing, Guiyuan Cao, Shibiao Wei, Zhihui Chen, Yunsong Di, Zhixing Gan

**Affiliations:** ^1^ Center for Future Optoelectronic Functional Materials, School of Computer and Electronic Information/School of Artificial Intelligence, Nanjing Normal University, Nanjing 210023, P. R. China.; ^2^ Joint International Research Laboratory of Information Display and Visualization, School of Electronic Science and Engineering, Southeast University, Nanjing 210096, P. R. China.; ^3^ Nanophotonics Research Center, Shenzhen Key Laboratory of Micro-Scale Optical Information Technology, Shenzhen University, Shenzhen 518060, P. R. China.; ^4^ Key Lab of Advanced Transducers and Intelligent Control System, Ministry of Education and Shanxi Province, College of Electronic Information and Optical Engineering, Taiyuan University of Technology, Taiyuan 030024, P. R. China.

## Abstract

Photonic synapses combining photosensitivity and synaptic function can efficiently perceive and memorize visual information, making them crucial for the development of artificial vision systems. However, the development of high-performance photonic synapses with low power consumption and rapid optical erasing ability remains challenging. Here, we propose a photon-modulated charging/discharging mechanism for self-powered photonic synapses. The current hysteresis enables the devices based on CsPbBr_3_/solvent/carbon nitride multilayer architecture to emulate synaptic behaviors, such as excitatory postsynaptic currents, paired-pulse facilitation, and long/short-term memory. Intriguingly, the unique radiation direction-dependent photocurrent endows the photonic synapses with the capability of optical writing and rapid optical erasing. Moreover, the photonic synapses exhibit exceptional performance in contrast enhancement and noise reduction owing to the notable synaptic plasticity. In simulations based on artificial neural network (ANN) algorithms, the pre-processing by our photonic synapses improves the recognition rate of handwritten digit from 11.4% (200 training epochs) to 85% (~60 training epochs). Furthermore, due to the excellent feature extraction and memory capability, an array based on the photonic synapses can imitate facial recognition of human retina without the assistance of ANN.

## Introduction

With the exponential growth in the demand for large-scale data processing, the traditional von Neumann architecture with complex hierarchical structures faces efficiency constraints and performance ceilings in handling complex tasks [1,2]. In comparison, neuromorphic computing allows for parallel processing and distributed storage, aligning more closely with the working principles of the human brain [3–5]. By simulating the connections and signal transmission between neurons, brain-inspired neuromorphic computing can achieve intelligent behaviors such as learning, memory, and perception, which is expected to break through data transmission bottlenecks [6–8]. The high degree of parallelism and integration make neuromorphic computing excel in handling huge amounts of data, demonstrating substantial potential in simulating artificial intelligence.

As a novel branch of neuromorphic devices, photonic synapses open the possibilities of noncontact optical writing strategy, offering promising opportunities for the development of high-performance neuromorphic vision sensors [9,10]. The primary functions of a photonic synapse include plastic photoelectric conductivity and photocurrent storage. During the past years, enormous efforts have been devoted to developing new architectures for photonic synapse. For example, Gholipour et al. [11] demonstrated a photonic synapse based on photosensitive amorphous metal-sulfide microfibers, which successfully emulate most synaptic functions of the central nervous system in biology. However, limited wavelength selectivity and inconspicuous synaptic plasticity constrain the application in neuromorphic computing. With band structure design, Wang et al. [12] demonstrated a photonic synapse based on CsPbBr_3_ and pentacene. The devices exhibited pronounced synaptic behaviors such as paired-pulse facilitation (PPF), short-term plasticity (STP), and long-term plasticity (LTP), along with multiple wavelength response from 365 nm to 660 nm. However, the device consumes high energy up to 1.4 × 10^−9^ J per event. In 2019, Wang et al. [13] demonstrated a MoS_2_-based artificial synapse transistor with low power consumption of about 80 pJ per spike. Furthermore, Li et al. [14] demonstrated a C_8_-BTBT-based organic ferroelectric synapse with even lower power consumption of about 0.0675 × 10^−18^ J per synaptic event. However, these devices usually lack the functionality of erasure.

Recently, 3-terminal synaptic devices with current-reset behavior are developed. For instance, Han et al. [15] demonstrated a synaptic transistor based on graphene/h-BN/CsPbBr_3_ quantum dots (QDs). The optical information can be erased when a negative gate voltage is applied. Li et al. [16] demonstrated a Cs_3_Bi_2_I_9_-based photonic synapse, where the trapped electrons could be electrically erased when applying a negative gate voltage. In addition to photo-memory based on charge trapping by defects or interfaces [17–19], the low migration rate of ions provides an extended capture time [20,21], facilitating the realization of synaptic plasticity. Meanwhile, photonic synapses based on ion modulation offer crucial inspiration for brain–computer interface technology. Very recently, Chen et al. [22] reported an organic electrochemical photonic synapse that enables light-gated ionic/electronic coupling. The device achieved high-density nonvolatile conductance states for neuromorphic computing and exhibited exceptional visible-light-responsive capability at low operating voltages. The nonvolatile current can be stepwise erased by the application of reverse gate electrical pulses. However, on the one hand, electrical erasing will introduce additional energy consumption, which is contrary to the low energy consumption target of neuromorphic computing. On the other hand, both excited and inhibitory post-synaptic current of biological synapses can be driven by a single type of external stimuli. The artificial photonic synapse that can be quickly erased by optical signal is highly desirable, but still to be developed.

In this work, a new architecture of self-powered photonic synapse is proposed and demonstrated based on the photon-modulated charging/discharging in multilayer structure of CsPbBr_3_ QDs/solvent/carbon nitrides (CNs). The photonic synapses show notable current hysteresis, successfully emulating most synaptic behaviors in terms of excitatory postsynaptic currents (EPSCs), PPF, STP, and LTP. Notably, the photonic synapses not only achieve optical writing/electrical erasing with low reverse bias voltage (110 mV) but also realize optical writing/optical erasing owing to the unique radiation direction-dependent photocurrent of the asymmetric device structure. This functionality greatly mitigates the power consumption pressure caused by traditional gate voltage-driven electrical erasing, making the CsPbBr_3_/solvent/CN-based photonic synapses particularly suitable for constructing artificial vision systems with ultralow power consumption. As a demonstration, 3-layer artificial neural network (ANN) was connected with the photonic synapses for recognizing handwritten digital images. The recognition accuracy of pre-processed handwritten digital images was improved from 13% to 85% due to the excellent contrast enhancement and denoising capabilities of the photonic synapses. Furthermore, we simulated the facial recognition by a 112 × 92 photonic synaptic arrays. The training and testing results of the model confirmed the efficient extraction capability of the array for key features in target images, although these images present various orientations or contain signal noise.

## Results and Discussion

**Fig. 1.**
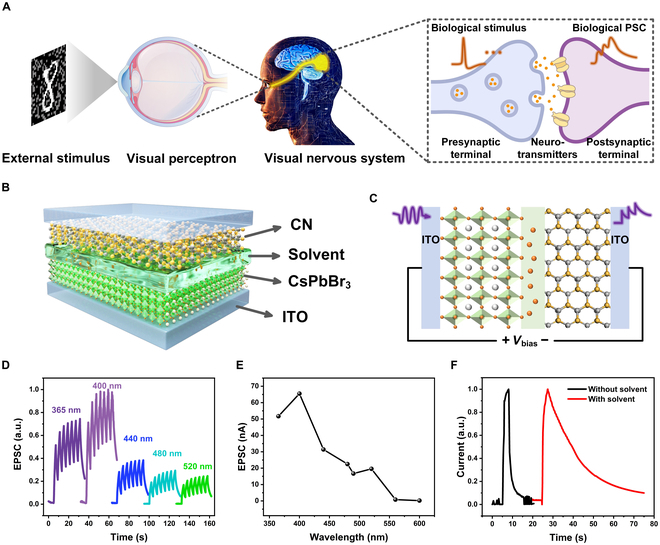
Design of the bioinspired photonic synapses. (A) Schematic illustration of the human neural visual perception system. (B) Structure of the CsPbBr_3_/solvent/CN-based artificial photonic synapses. (C) Schematic diagram of the light-stimulated photonic synapses and the direction of bias voltage. (D) Wavelength-dependent photocurrent of the photonic synapses based on CsPbBr_3_/solvent/CN when CsPbBr_3_ is illuminated (7.5 mW/cm^2^ and *V*_bias_ = 0 V). (E) Dependence of photocurrent on wavelength. (F) Photocurrent of the photonic synapses with and without organic solvent under 365-nm light illumination at zero bias.

As depicted in Fig. 1A, the eye serves as the primary sensory organ of the human visual system, responsible for perceiving and transmitting nearly 80% of external environmental stimulus to the brain for processing [23–25]. Synapses are the connection points between different neurons, acting as bridges for communication and signal transmission. Once a synapse receives a biological spike, the neurotransmitters will be released from vesicles in the presynaptic membrane and delivered across the synaptic gap to receptors at the postsynaptic terminal, leading to a change in the amplitude of the EPSC [26–28]. This intricate process involves the modulation of ion channels by neurotransmitters, where synaptic weight is defined as the strength of connections between synapses. The weights that change with different synaptic activities are referred as synaptic plasticity, which is the physiological basis of human learning and memory behavior [29]. Therefore, it is very crucial to endow photodetector with optical plasticity for the construction of a photonic synapse.

The device structure of photonic synapse based on CsPbBr_3_/solvent/CN is schemed in Fig. 1B. The transmission electron microscopy (TEM) image, high-resolution (HR) TEM image of the CsPbBr_3_ QDs, and the scanning electron microscopy (SEM) image of CN are shown in Fig. S1. Figure S2 shows the x-ray diffraction (XRD) patterns of them. The diffraction peaks are located at 14.91°, 21.32°, 30.41°, 37.58°, and 43.62°, which can be assigned to the (100), (110), (200), (211), and (202) planes of CsPbBr_3_ QDs cubic phase [30,31]. The CN shows 2 characteristic peaks at 12.96° and 27.52°, which are corresponded to the (100) and (002) crystal planes [32,33]. The absorption and photoluminescence (PL) spectra of CsPbBr_3_ QDs and CN are shown in Fig. S3. The CsPbBr_3_ QDs show a narrow PL band at about 520 nm, while the CN exhibits a relative broadband PL peaking at 450 nm. As shown in Fig. S4 and Table S1, time-resolved PL (TRPL) decay spectra of CsPbBr_3_ and CN show that their carrier lifetimes are about 31.55 and 3.47 ns, respectively. The transparent region between CsPbBr_3_ and CN represents the organic solvent toluene. Due to the ionic nature of CsPbBr_3_, it is reasonable that the solvent contains a considerable amount of bromide (Br) ions detached from the structure of the CsPbBr_3_ QD lattice, which is confirmed by inductively coupled plasma atomic emission spectrometry test. A photo and a fluorescent photo of the 2-terminal synaptic devices based on CsPbBr_3_/solvent/CN are shown in Fig. S5.

Figure 1C illustrates the artificial photonic synapses driven by light stimulation, corresponding to the generation of action potentials in biological synapses. The direction of the bias voltage is also specified, providing a reference for the following discussion on the mechanism of the synaptic effect. Figure 1D shows the photocurrent of the device excited at different wavelengths at zero bias when CsPbBr_3_ is illuminated. The photonic synapses exhibit notable positive photocurrent, while the photocurrent gradually weakens when the wavelength of light pulses increases from 440 to 520 nm (Fig. 1E). It is worth noting that the photocurrent slowly decays after ceasing the light pulse, implying the existence of a temporary photo-storage. As shown in Fig. S6, the photocurrent of the photonic synapses is reversed when CN is illuminated, indicating the directional dependence of the photocurrent. Unless otherwise specified, the illumination is directed towards the CsPbBr_3_ side. Figure 1F shows the photocurrent decay of the photonic synapse based on CsPbBr_3_/solvent/CN under 365 nm light stimulus at zero bias. After removal of the light pulse (duration = 3 s, 7.55 mW/cm^2^), the photocurrent lasts for about 50 s. We would like to emphasize that the persistent photocurrent strongly depends on the organic solvent layer. Without the solvent layer, photocurrent decay of the device based on CsPbBr_3_/CN only lasts for 7 s. The solvent layer plays the role similar to electrolyte in electrochemical batteries, which blocks the direct recombination of photogenerated electrons and holes. Moreover, the Br^−^ ions distributing in the solvent can capture and store the photogenerated holes at the interfaces. These 2 effects jointly lead to the persistent photocurrent that can simulate the EPSC.

**Fig. 2.**
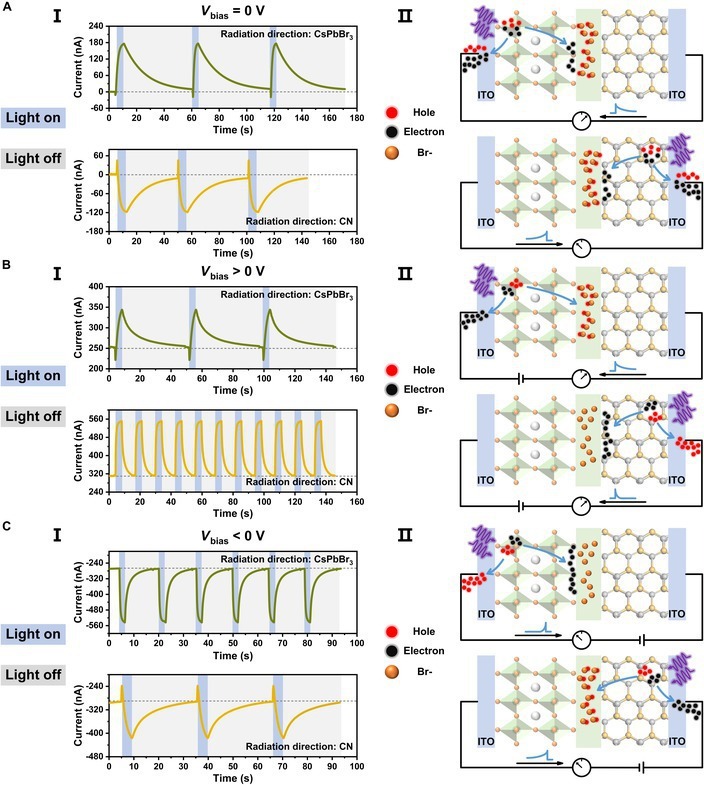
Persistent photocurrent and underlying mechanisms. (A to C) Time-resolved photocurrent (I) and charge-trapping state (II) of the device under zero bias voltage (A), forward 0.1-V bias voltage (B), and reverse −0.1-V bias voltage (C).

To gain more insight into the persistent photocurrent, in-depth photoelectrical tests were conducted. Figure 2A to C show the time-resolved photocurrent curves of the device under different radiation directions and bias voltages. At zero bias, abnormally, the photocurrent direction depends on the light radiation direction (Fig. 2AI). The device displays a positive persistent photocurrent when the light stimulus is applied to the CsPbBr_3_ side, while a negative photocurrent is observed when the light is illuminated on the CN. As schemed in Fig. 2AII, when the CsPbBr_3_ QDs are excited, both electrons and holes are generated. They uniformly diffuse outward due to the concentration gradient without net current. However, when the holes approach the solvent, they are captured by the negatively charged Br^−^ ions. Therefore, there is a net diffusion of negative charges at the direction opposite to solvent (left in the scheme). According to the designated direction, a positive current is observed. Similarly, when the light is irradiated on the CN, the photogenerated holes are captured by the Br^−^ ions in the solvent. Thus, there is a net diffusion of negative charges along the right direction in the scheme. As a result, the photocurrent is negative. Accordingly, the multilayer device containing semiconductor and solvent is analog to a photo-electro-battery combing the photo-electro-conversion and charge storage. When the light stimulation is stopped, the charges deposited in the solvent are gradually released, thus generating a persistent photocurrent.

When a positive voltage is applied, due to the electrical field-driven drift of carriers, only positive current is observed no matter what direction of the irradiation (Fig. 2B). However, the decay time of the photocurrent depends on the irradiation direction. If the CNs are excited, most of the photogenerated holes cannot be captured by the solvent due to the drift on the direction opposite to solvent. As a result, the persistent photocurrent almost disappears, whereas, if the CsPbBr_3_ QDs are excited, persistent photocurrent retains. When a negative voltage is applied, the photocurrents are negative. The negative persistent photocurrent is mainly observed by irradiating on the CN. The photo-modulated charging/discharging mechanism is further confirmed by the current–voltage (*I*–*V*) characteristic curve (Fig. S7).

**Fig. 3.**
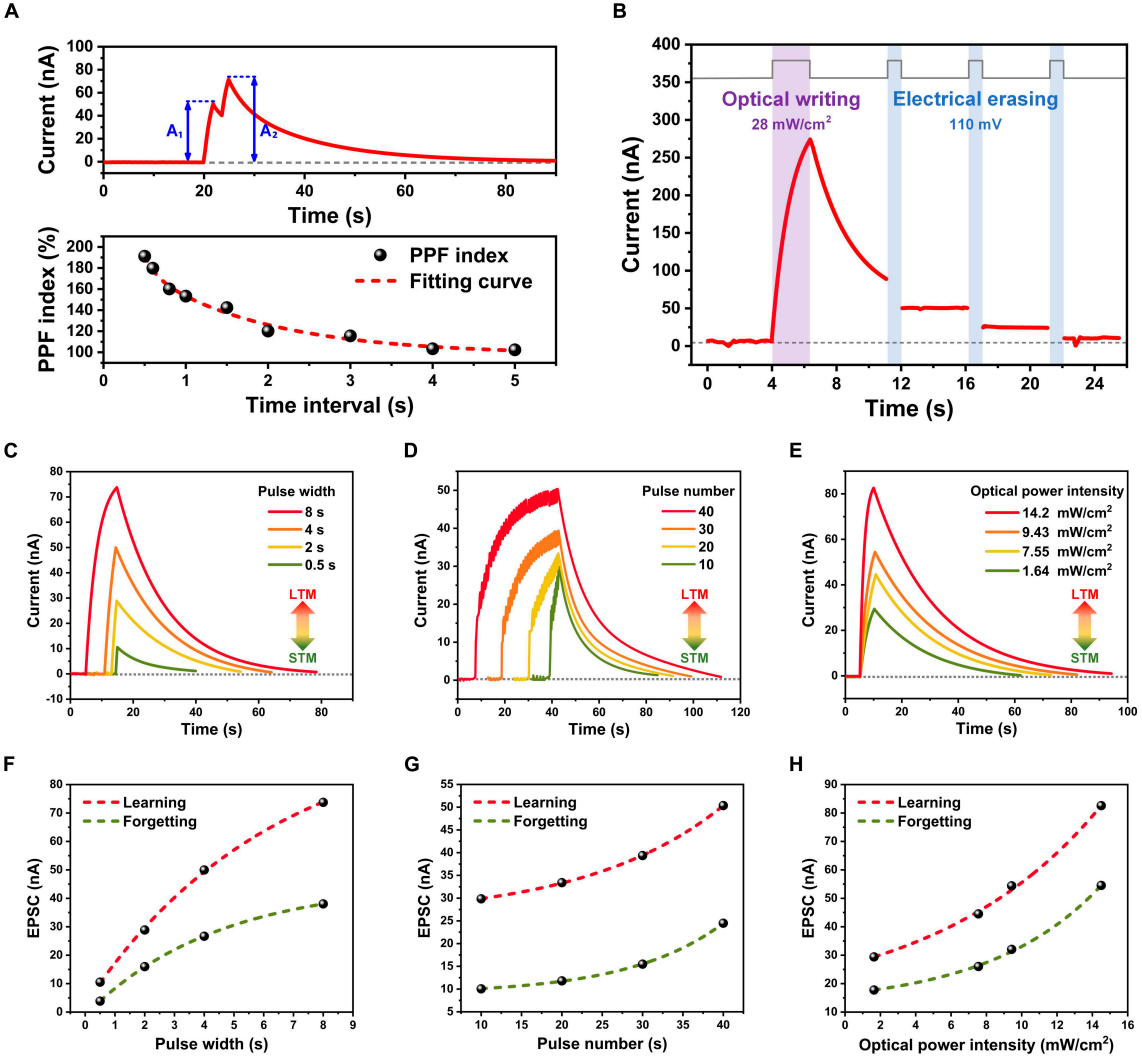
Optical synaptic plasticity of the device for mimicking bio-synapses. (A) Top: EPSC evoked by a pair of light pulses with 1.5-s interval (365 nm, 7.55 mW/cm^2^, *V*_bias_ = 0 V). Bottom: PPF index as a function of different time intervals. (B) Optical writing and electrical erasing process of the device under 365-nm illumination at zero bias. (C to E) Transition process from STP to LTP measured under different stimulation conditions by changing the pulse width (C), pulse number (D), and pulse intensity (E). (F to H) Relationship between EPSC and pulse width (F), pulse number (G), and pulse intensity (H).

According to the novel photo-modulated charging/discharging mechanism, it is reasonable that photoconductivity of the CsPbBr_3_/solvent/CN device depends on the irradiation condition. When the dose of photo-stimulus increases, the photoconductivity increases and memory time elongates. The reconfigurable photoconductivity suggests that the CsPbBr_3_/solvent/CN device can play the role of a photonic synapse. The synaptic plasticity in biology refers to the characteristics of relative changes in the morphology, function, strength, and efficiency of synapses [34,35]. It can be categorized into STP and LTP according to the duration of memory [36,37]. STP plays a pivotal role in processing and decoding temporal information in the human brain. PPF is an essential characteristic of STP, denoting the rate of enhancement in postsynaptic current between 2 consecutive stimulus pulses [38–40]. As shown in the upper panel of Fig. 3A, a pair of light pulses with identical pulse widths and an interval of 1.5 s were applied to the CsPbBr_3_ side of the device at zero bias. The second EPSC value (*A*_2_) is obviously higher than the first one (*A*_1_) because the photo-generated carriers in the device do not decay to the initial level before the arrival of the next pulse due to the photo-storage effect. As shown in Fig. 3A, the fitting curve suggests that the PPF index (*A*_2_/*A*_1_ × 100%) follows a double exponential function of the light pulse intervals. Shorter time intervals result in a more pronounced current enhancement, which is consistent with the observations in biological synapses [18,41,42].

Notably, when negative electrical pulses are applied to the device after the light pulse is removed, the charge carriers captured by the solvent could be electrically released, restoring the nonvolatile photocurrent to its initial state. The result shown in Fig. 3B demonstrates the device’s capability of optical writing and electrical erasing, enhancing the flexibility of information storage and weight updating. The biologic synapses can transform from STP to LTP through rehearsal learning, such as improving the spike duration, number, and intensity [43,44]. Here, the photonic synapse based on CsPbBr_3_/solvent/CN successfully simulates the transition process from STP to LTP by adjusting the pulse width, pulse number, and light intensity. Figure 3C to E shows the postsynaptic current curves of the device measured under different stimuli. The dependences of EPSC and decay time on the spike conditions are shown in Fig. 3F to H. The EPSC of the device increases dramatically as the light pulse duration increases from 0.5 s to 8 s. The spike number and optical power intensity also have a similar impact on the learning and forgetting process of the device. More number of spikes or higher light intensity leads to enhanced EPSC levels. Higher EPSC levels always require longer decay times to return to initial levels. As shown in Fig. S8, under optical spike of 25 s duration and 25 mW/cm^2^ intensity, the photocurrent retention time can be extended to over 300 s. These results demonstrate that the photonic synapse possesses exceptional capabilities for perceiving and storing different optical information. Additionally, the photonic synapses can imitate the learning–forgetting–relearning behavior (Fig. S9). Spike frequency-dependent EPSC is also observed in our photonic synapses (Fig. S10).

**Fig. 4.**
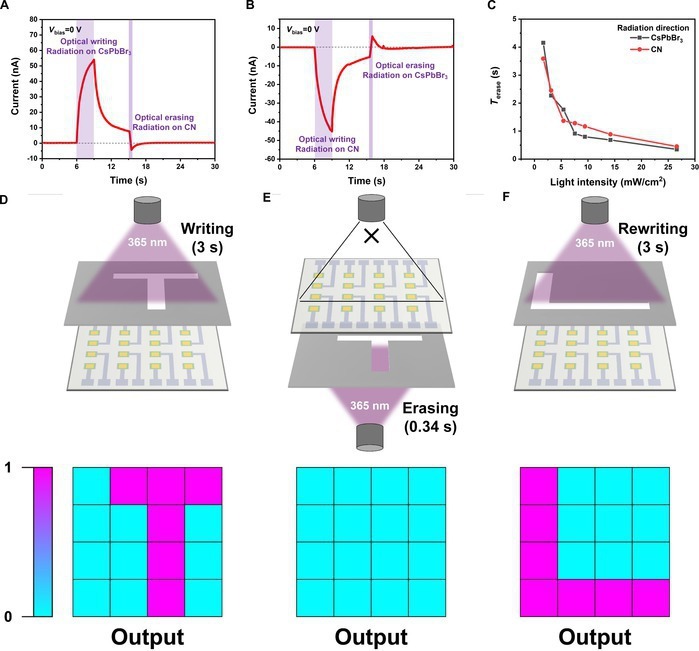
Optical erasing capabilities of the photonic synapse. (A and B) Process of optical writing/optical erasing when CsPbBr_3_ (A) or CN (B) is illuminated under zero bias (7.55 mW/cm^2^). (C) Relationship between *t*_erase_ and erasing pulse intensity. (D) A T-shaped pattern of light is projected onto a 4 × 4 synaptic array. (E) The T-shaped photocurrent is rapidly erased by the application of reverse illumination. (F) An L-shaped pattern of light is projected onto the synaptic array after optical erasing.

More importantly, the photonic synapses possess optical writing/optical erasing functionality by utilizing the radiation direction-dependent photocurrent. As shown in Fig. 4A and B, regardless of whether the light illuminates on CsPbBr_3_ or CN, nonvolatile current can be rapidly erased and restored to its initial state by the light pulses on the opposite side. As the erasing light intensity increases, the erasing time (*t*_erase_) progressively shortens with a *t*_erase_ (<2 s) at an intensity of 26.8 mW/cm^2^ (Fig. 4C). As a demonstration, a prototype synaptic array with 4 × 4 pixels was prepared to verify the optical erasing performance (Fig. S11). The memory ability and hysteresis current of optoelectronic synapses mean that they cannot rapidly switch between different recognition tasks. Before initiating the next recognition task, waiting for complete photocurrent decay is time consuming. Herein, the optical writing and erasing respond to same optical stimuli. The nonvolatile photocurrent can be quickly reset to its initial state by reversing the stimulus direction, which is lacking in other photonic synapses (Fig. 4D and E and Note S1). A comprehensive comparison of synaptic functionalities with other photonic synapses is shown in Table S2, showing that the CsPbBr_3_/solvent/CN-based photonic synapses exhibit notable advantages in terms of power consumption, erasure capability, and PPF index. Moreover, as shown in Fig. S12, the device retains its hysteresis current characteristics after storage in the ambient environment for 6 months.

**Fig. 5.**
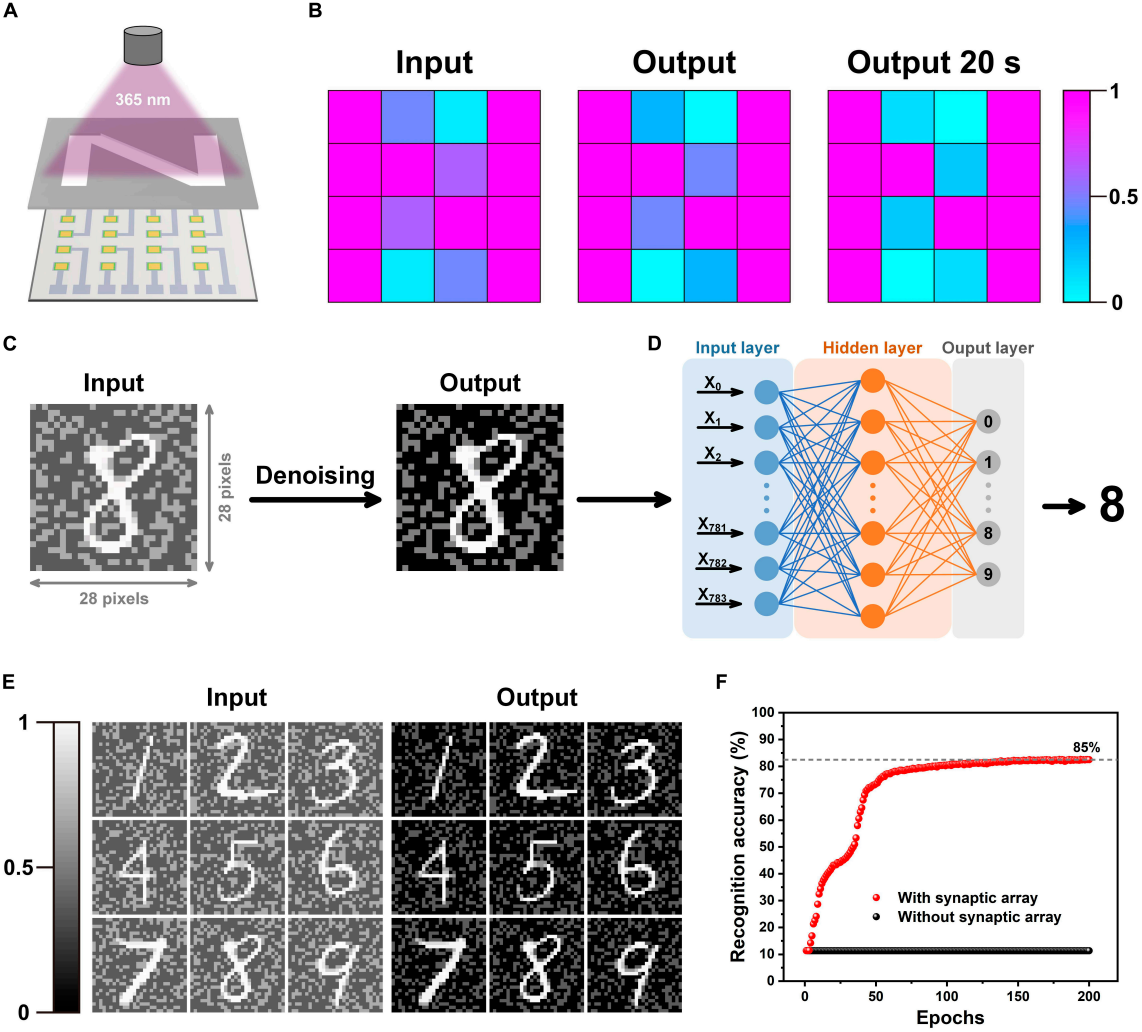
Image preprocessing capabilities of the photonic synapse. (A) Schematic illustration for measuring the N-type patterned light. (B) Image contrast enhancement process based on the 4 × 4 array. (C and D) The 28 pixel × 28 pixel handwritten digit image with added background noise is preprocessed (C) and input into 3-layer ANNs (D) for recognition. (E) Contrast enhancement and denoised effect of handwritten digits after pre-processed by the photonic synapses. (F) Comparisons of the image recognition accuracy at different epochs with and without preprocessing by the synaptic array.

The integrated functions of photo-electrical conversion and temporal storage endow the photonic synapse’s ability for image perception and preprocessing simultaneously. Image preprocessing stands as a pivotal stage in the field of computer vision and image analysis [45,46]. Reasonable image preprocessing serves to effectively diminish noise, accentuate crucial features, and maximize the quality of input images. Due to the outstanding capabilities in optical information perception and storage, the photonic synapse can realize the image preprocessing functions on the hardware level. As shown in Fig. 5A, patterned illumination was applied to the synaptic array by an N-type shadow mask. The photocurrent measured from each pixel of the photonic synaptic array after illumination is shown in Fig. 5B, displaying a relatively distinct N-shaped distribution of photocurrents. However, some remaining noise pixels will affect the observation of the pattern. This problem can be self-alleviated after a short delay, which is attributed to the faster decay of EPSC under weaker light stimulation. As shown in the right panel of Fig. 5B, the contrast of the pattern is enhanced after 20 s, indicating the great capacity of the photonic synapse in terms of contrast enhancement and noise reduction.

Based on the above results, a multilayer perceptron (MLP) was constructed (software algorithm) for simulating the digit recognition task of the Modified National Institute of Standards and Technology (MNIST) handwriting image dataset (Note S2). Figure 5C illustrates the process of recognizing digital images through ANNs after preprocessing by the photonic synapse. The input is a digital image with added background noise, where the background noise is randomly generated within the normalized range of 0.4 to 0.6. After preprocessed through the photonic synapse, the high-quality reduction of background noise enhances the digital characteristics of the output image. Figure 5D shows the schematic diagram of the ANNs, which consists of an input layer (784 neurons corresponding to 28 × 28 pixels of the input image), a hidden layer (25 neurons), and an output layer (10 neurons corresponding to the numbers 0 to 9). Typical examples of preprocessing on the images with background noise through photonic synapses are shown Fig. 5E. The features of each number are dramatically enhanced, which results in a higher image recognition accuracy with fewer epochs in digit recognition tasks. As shown in Fig. 5F, inputting the preprocessed image into the simple ANN can achieve a recognition rate of 80% within about 60 training epochs. However, the recognition rate is only 11.4% after 200 training epochs when images are recognized directly without preprocessing.

**Fig. 6.**
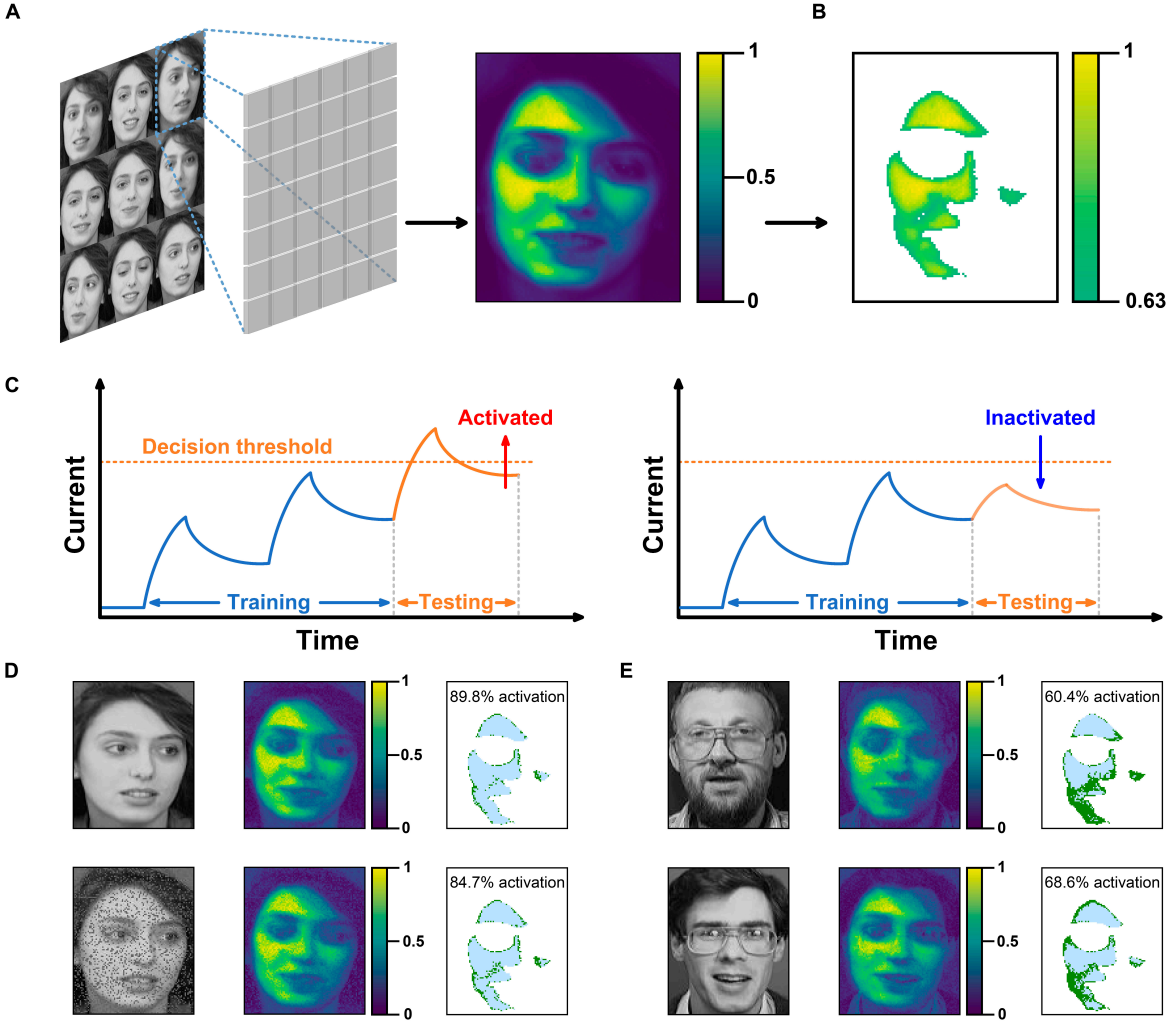
Artificial retina based on the photonic synapses for face recognition. (A) Illustration of the model training of the photonic synaptic array. (B) Facial features extracted after training. (C) Scheme of the training, testing, and threshold currents of the synaptic unit. Units with current higher than the threshold are classified as active, while units with current lower than the threshold are classified as inactive. (D and E) Results of facial recognition tests. The array exhibits a high activation rate for the target facial image (D), even when these images have added signal noise, but shows a low activation rate for facial images that are different to the training one (E). Credit: Photographs of faces, AT&T Laboratories Cambridge.

Moreover, the photonic synapses can also imitate the visual neurons to conduct recognition tasks without the assistance of ANN. To demonstrate this function, a neuromorphic visual sensor (NVS) containing a 112 × 92 synaptic array was designed to mimic the face recognition of the human visual system. The model was trained and tested using the database of faces from AT&T Laboratories Cambridge, which consists of multiple human face images with different features. As shown in Fig. 6A, 9 grayscale images of female faces containing different facial orientations, expressions, and emotions were used as input to train the feature store ability of the NVS. In our simulations, facial features are represented by the light intensities. The synaptic unit and image pixel are in one-to-one correspondence. Thus, each synaptic unit can obtain the corresponding EPSC according to the spike intensity–EPSC function in Fig. 3H. For example, the grayscale intensity of the cheek is higher relative to the hair or eyes due to the stronger light reflections, suggesting that the NVS generates higher memory currents in the vicinity of the facial features (cheeks, forehead, chin). Moreover, due to the PPF effect of the synapses, the high current of these pixels will ascend faster with the continuous increment of training iterations. Therefore, facial features will be extracted after training on 9 images, as shown in Fig. 6B. The facial features are temporally stored in the NVS. By setting a threshold current (Fig. 6C), the memory currents are used as the facial feature outline for constructing the recognition model.

In the testing process, a decision threshold is assigned to each unit in the NVS. When a testing pixel is similar to the training one, the current of the corresponding unit exceeds the decision threshold due to the PPF effect, and the unit reaches an activated state, vice versa. Hence, if facial images from the same woman are input into NVS, almost all the units are activated. Otherwise, a portion of units will stay in an inactive state. Here, 4 example facial images are employed to validate the recognition effect of the constructed face recognition model. As shown in Fig. 6D, when the test image came from the target facial image, about 90% of the units are activated. We also tested target facial images with added noise, and the NVS can still exhibit an activation rate up to about 85%. However, when tested on facial images of other men, the activation rate is only around 60% (Fig. 6E). Furthermore, the NVS can efficiently extract key features of faces with different orientations and expressions (Fig. S13). All these results collectively confirm the excellent feature extraction capability of the NVS based on our photonic synapses.

## Conclusion

In conclusion, we propose a photon-modulated charging/discharging mechanism for self-powered photonic synapses. Synaptic behaviors such as EPSC, PPF, STP, and LTP were successfully emulated by the photonic synapses based on CsPbBr_3_/solvent/CN. Notably, in addition to electrical erasing, photonic synapses also possess the novel optical writing/optical erasing ability owing to the radiation direction-dependent EPSC. This functionality substantially reduces the energy consumption, offering new insights for constructing high-performance neuromorphic devices in the future. Moreover, the exceptional synaptic plasticity endows photonic synapses with remarkable contrast enhancement and noise reduction capabilities. After being pre-processed by photonic synapses, the recognition of handwritten digit images by ANN possesses high accuracy up to 85% within about 60 training epochs. Moreover, a facial recognition system based on the synaptic arrays was also constructed without the use of ANN, which can effectively extract the key features of facial images.

## Methods

Detailed information about the methods used in this work is available in the supplementary materials.

## Data Availability

All data are available from the authors upon request.

## Supplementary Materials

Supplementary 1 Supplementary Methods Notes S1 and S2 Figs. S1 to S13 Tables S1 and S2 References
