# Supplementary material for: Self-Powered Photonic Synapses with Rapid Optical Erasing Ability for Neuromorphic Visual Perception
Source: Research (Wash D C). 2024 Nov 7;7:0526. doi: 10.34133/research.0526 (PMC11542608; doi:10.34133/research.0526)
Supplement: Supplementary 1 — Supplementary Methods Notes S1 and S2 Figs. S1 to S13 Tables S1 and S2 References [file research.0526.f1.docx]

**Supporting Information**

Self-powered photonic synapses with rapid optical erasing ability for neuromorphic visual perception

Mingchao Li,^1#^ Chen Li,^2#^ Kang Ye,^1#^ Yunzhe Xu,^2^ Weichen Song,^2^ Cihui Liu,^1^ Fangjian Xing,^1^ Guiyuan Cao,^3^ Shibiao Wei,^3^ Zhihui Chen,^4^ Yunsong Di,^1*^ and Zhixing Gan^1,*^

1 Center for Future Optoelectronic Functional Materials, School of Computer and Electronic Information/School of Artificial Intelligence, Nanjing Normal University, Nanjing 210023, P. R. China

2 Joint International Research Laboratory of Information Display and Visualization, School of Electronic Science and Engineering, Southeast University, Nanjing 210096, P. R. China

3 Nanophotonics Research Center, Shenzhen Key Laboratory of Micro-Scale Optical Information Technology, Shenzhen University, Shenzhen 518060, P. R. China

4 Key Lab of Advanced Transducers and Intelligent Control System, Ministry of Education and Shanxi Province, College of Electronic Information and Optical Engineering, Taiyuan University of Technology, Taiyuan 030024, P. R. China

#These authors contributed equally to this work.

*Corresponding authors’ e-mail addresses.

zxgan@njnu.edu.cn (ZX Gan)

diyunsong@njnu.edu.cn (YS Di)

**Methods**

**Materials.**

All the reagents were used directly without further purification. Caesium carbonate (Cs_2_CO_3_), 1-octadecene (ODE), oleylamine (OAM), and Polystyrene (PS) were bought from Aladdin Ltd. Oleic acid (OA) was bought from Sigma Aldrich. Lead bromide (PbBr_2_) and Melamine were bought from Macklin. Toluene was bought from Nanjing Chemical Reagent. Anhydrous ethanol (99.7%) and deionized water were obtained from local sources.

**Synthesis of CsPbBr_3_ QDs.**

The CsPbBr_3_ QDs was synthesized using a hot-injection method. Firstly, cesium oleate (Cs-oleate) precursor solution was prepared by dissolving Cs_2_CO_3_ (0.25 g), OA (1 mL), and ODE (25 mL) in a 50 mL three-neck flask and dried for 1 h under vacuum conditions at 120 °C, then heated up to 150 °C at N_2_ atmosphere. The product was collected once all Cs_2_CO_3_ powder was dissolved, and the solution became clear. Next, PbBr_2_ (0.138 g), OA (1 mL), and OAM (1 mL) were dissolved in 10 ml of ODE in a 100 mL three-neck flask. The mixture was stirred under vacuum conditions and heated to 120 ℃. After stirred for about 50 minutes, N_2_ was introduced, and the mixture was further heated to 170 ℃. Then, 0.8 mL of Cs-oleate solution (preheated to 100 °C) was rapidly injected into the mixture upon all PbBr_2_ powders were dissolved. After 5 s, the three-neck flask was cooled down by an ice water bath. Subsequently, the obtained crude solution was centrifuged at 8000 rpm for 5 minutes. Then the precipitate was collected and dissolved in toluene (5 mL) after discarding the supernatant. The solution was centrifuged at 8000 rpm for 5 min again, and the supernatant was the CsPbBr_3_ QDs.

**Synthesis of carbon nitride.**

Carbon nitride (CN) was prepared by thermal condensation polymerization of melamine. In detail, analytical grade melamine powder (5 g) was pressed into a ceramic boat and then placed in the middle area of the reaction chamber. The melamine powder was heated to the preset temperature of 700 °C for 2 h with a heating rate of 3 °C/min. The ceramic boat was naturally cooled down to room temperature when the reaction was completed. Finally, the resultant yellow agglomerates were milled into powder in an agate mortar.

**Structural and Optical Characterization:**

The morphology of the samples was observed by a JEM-2100 plus transmission electron microscope (TEM) and a JSM-6700F scanning electron microscope (SEM). The X-ray diffraction (XRD) measurements were performed on a Bruker D8 diffractometer using Cu Kα radiation. PL spectra were obtained using a micro-fluorescence device equipped with a Maya 2000 Pro high-sensitivity spectrometer (Ocean Optics), and the excitation source was a 405-nm laser with either pulsed or CW modes. UV-vis optical absorption spectra were obtained by using Shimadzu UV-2600 spectrophotometer.

**Device Fabrication and Characterization.**

Two pieces of glass substrates coated with indium tin oxide (ITO) were sonicated in anhydrous ethanol and deionized water for 30 minutes, followed by placement in a drying oven and heating at 80 °C for 15 minutes to prevent the substrates from being contaminated by residual solvents. Polystyrene (PS) solution was prepared by adding PS pellets (400 mg) to toluene solution (10 mL). Traceless tape was applied to the central portion of the two cleaned ITO glass pieces. Then, 0.2 mL of PS solution was transferred onto the ITO glass using a pipette and spin-coated at a rotation speed of 3000 rpm for 1 minute. The tape was then removed after the solution solidifies. Subsequently, 0.2 mL of CsPbBr_3_ QDs was dispensed onto the conductive portion of the ITO glass using a pipette, resulting in the formation of a viscous thin film with toluene. CN dispersion was prepared by adding CN powders (400 mg) to toluene solution (5 mL). Following the similar procedure, 0.2 mL of CN dispersion was also dispensed onto the conductive portion of another ITO glass using a pipette. Without waiting for the toluene to volatilize, the two pieces of ITO containing CsPbBr_3_ QDs film and CN film were directly attached by clips and encapsulated by polymer to prepare the CsPbBr_3_ QDs/solvent/CN-based photonic synaptic device.

The I-V curves and photocurrent were measured by a dual-channel Keithley 2450 source-meter. A picosecond supercontinuum laser (YSL, SC-PRO-M) with a repetition frequency of 20 MHz was used to investigate the wavelength-dependent photocurrent of the photonic synapses. The incident power is calibrated by an optical power meter. Pulsed light was output by an LED, which was powered and regulated by a signal generator.


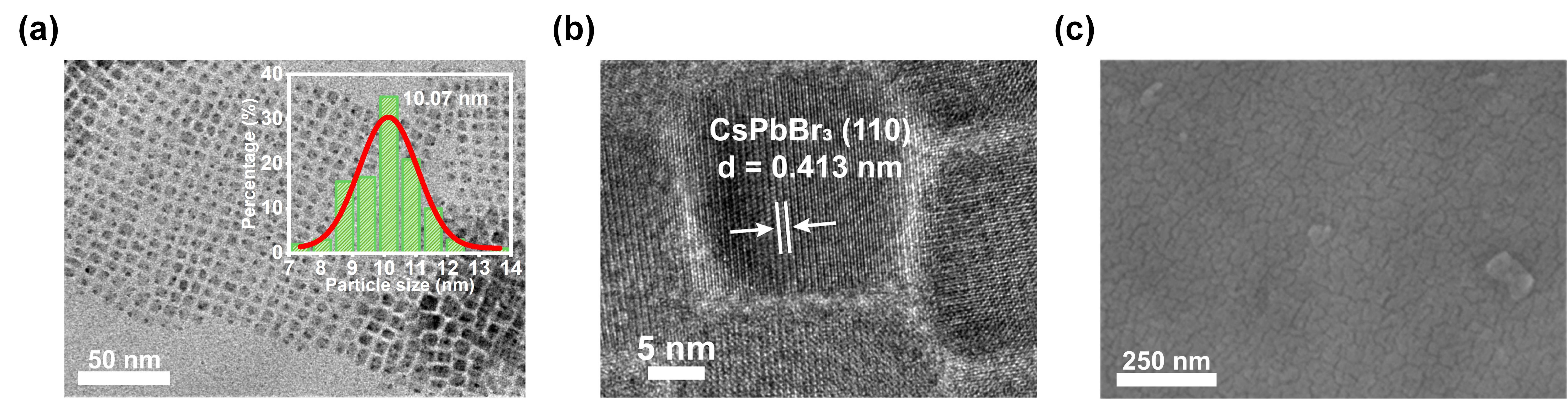


Figure S1. (a) Transmission electron microscopy (TEM) image of the CsPbBr_3_ QDs. The inset shows the size distribution. (b) HRTEM image of the CsPbBr_3_ QDs. (c) Scanning electron microscopy (SEM) image of the CN film.

The sizes of CsPbBr_3_ QDs vary between 7 and 14 nm, and the most probable size is about 10.07 nm. The CsPbBr_3_ QDs are crystalline nanocubes exhibiting a distinct interplanar spacing of 0.413 nm corresponding to the (110) crystal planes, while CN are 2D micro-sheets.





Figure S2. X-ray diffraction pattern of the CsPbBr_3_ QDs (top) and CN (bottom).


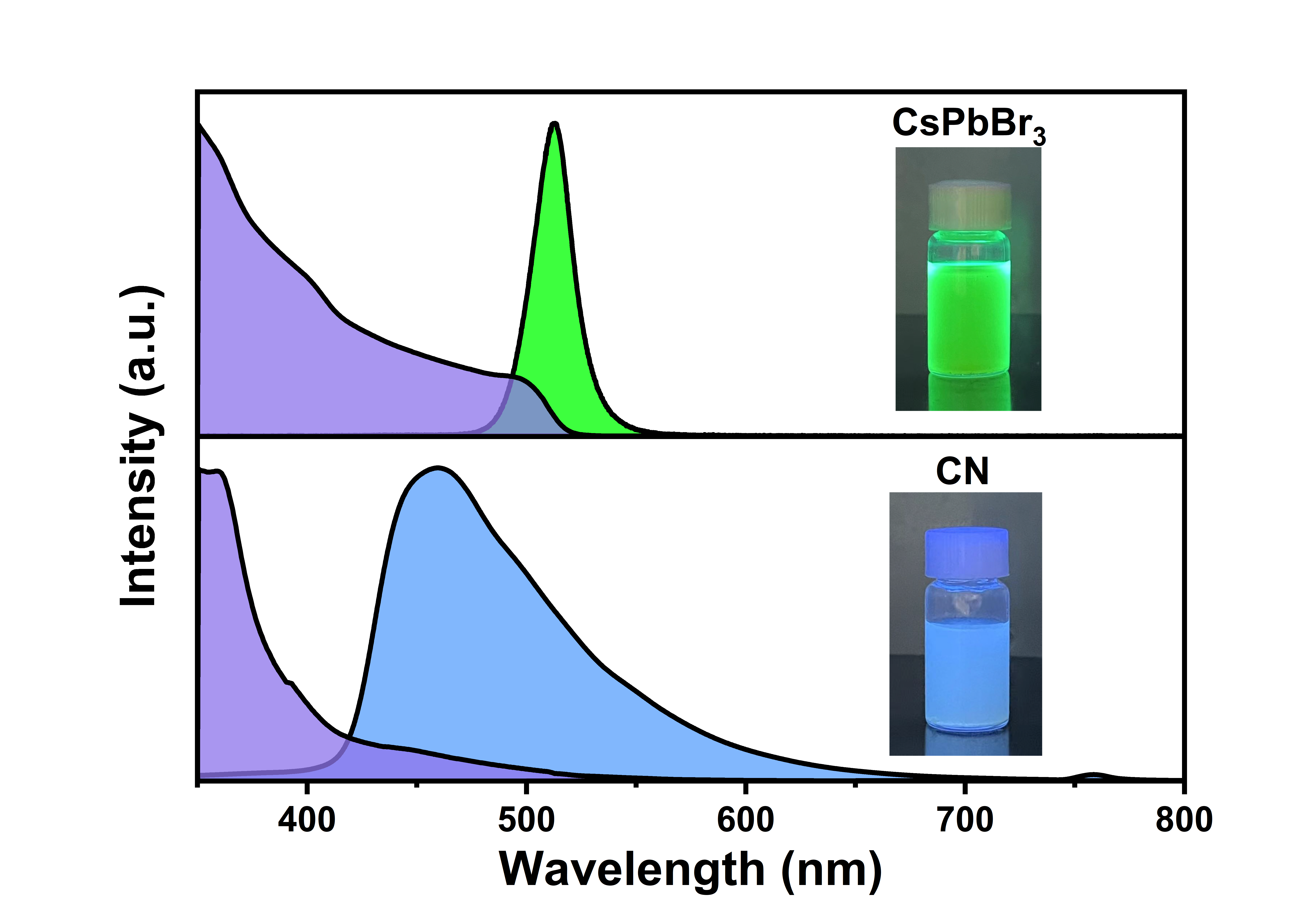


Figure S3. Normalized UV-vis absorption and PL spectra of CsPbBr_3_ QDs (top) and CN (bottom). Insets: fluorescence images of the CsPbBr_3_ QDs and CN solutions under 365 nm light illumination.


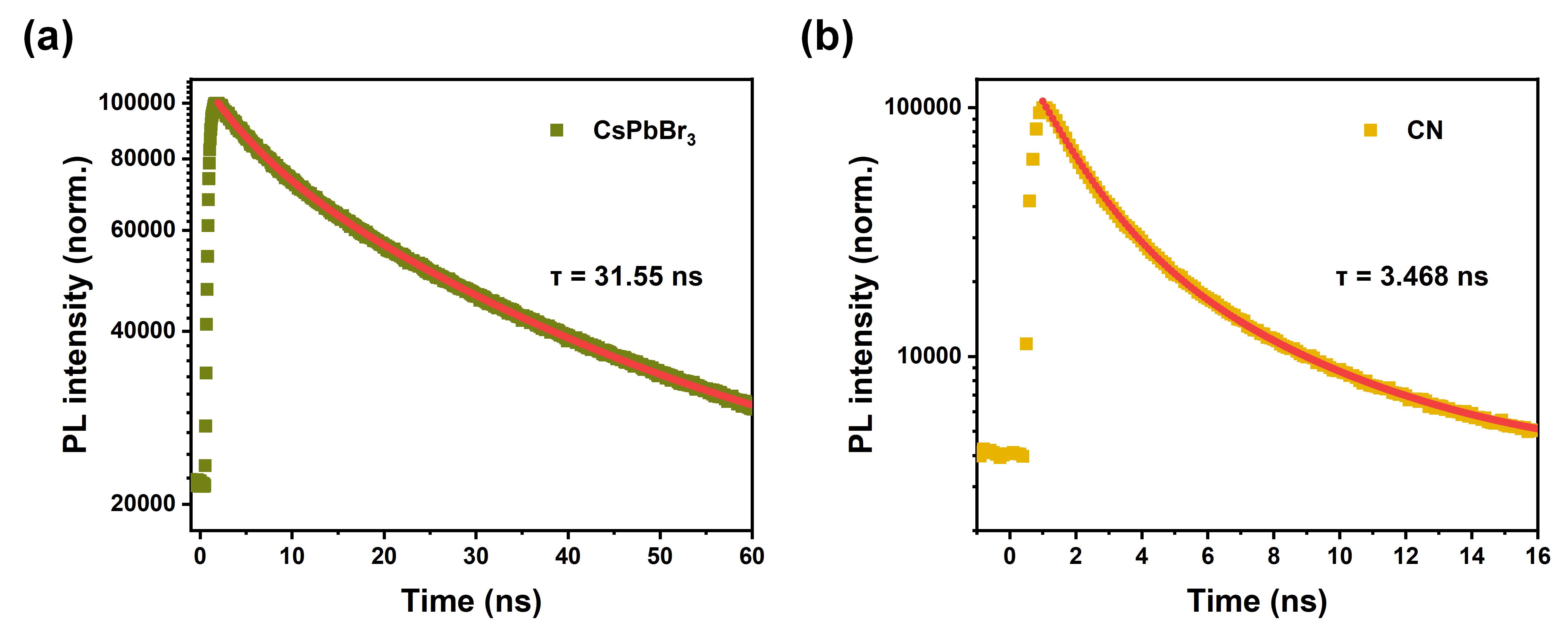


Figure S4. Time-resolved photoluminescence (TRPL) spectroscopy of CsPbBr_3_ (a) and CN (b), respectively.

The PL decay curves of CsPbBr_3_ and CN are both fitted by biexponential functions:

$$I\left( t \right)=A_{1}\exp\left( -\frac{t}{\tau_{1}} \right)+A_{2}\exp\left( -\frac{t}{\tau_{2}} \right)$$

where A_1_ and A_2_ are the fitting coefficients, τ_1_ and τ_2_ are the characteristic lifetimes of the two PL decay components, respectively. The fitted lifetime parameters are listed in Table R1. The average PL lifetimes of CsPbBr_3_ and CN are calculated to be about 31.55 ns and 3.468 ns, respectively.

Table S1. Fitted Lifetime Parameters Corresponding to the Time-Resolved PL Decay Curves

| Materials | A_1_ | τ_1_ (ns) | A_2_ | τ_2_ (ns) | $\bar{\text{τ}}=\frac{A_{1}{\tau_{1}}^{2}+A_{2}{\tau_{2}}^{2}}{A_{1}\tau_{1}+A_{2}\tau_{2}}$ |
| --- | --- | --- | --- | --- | --- |
| CsPbBr_3_ | 14177.27952 | 5.62609 | 62287.81816 | 32.57085 | 31.55 ns |
| CN | 67071.50485 | 1.36917 | 32188.9572 | 4.73287 | 3.468 ns |





Figure S5. (a) Fluorescence image of the CsPbBr_3_/solvent/CN-based photonic synapse, where the marked red square region is the PS insulating layer. (b) Fluorescence images of CN film and CsPbBr_3_ QDs film.


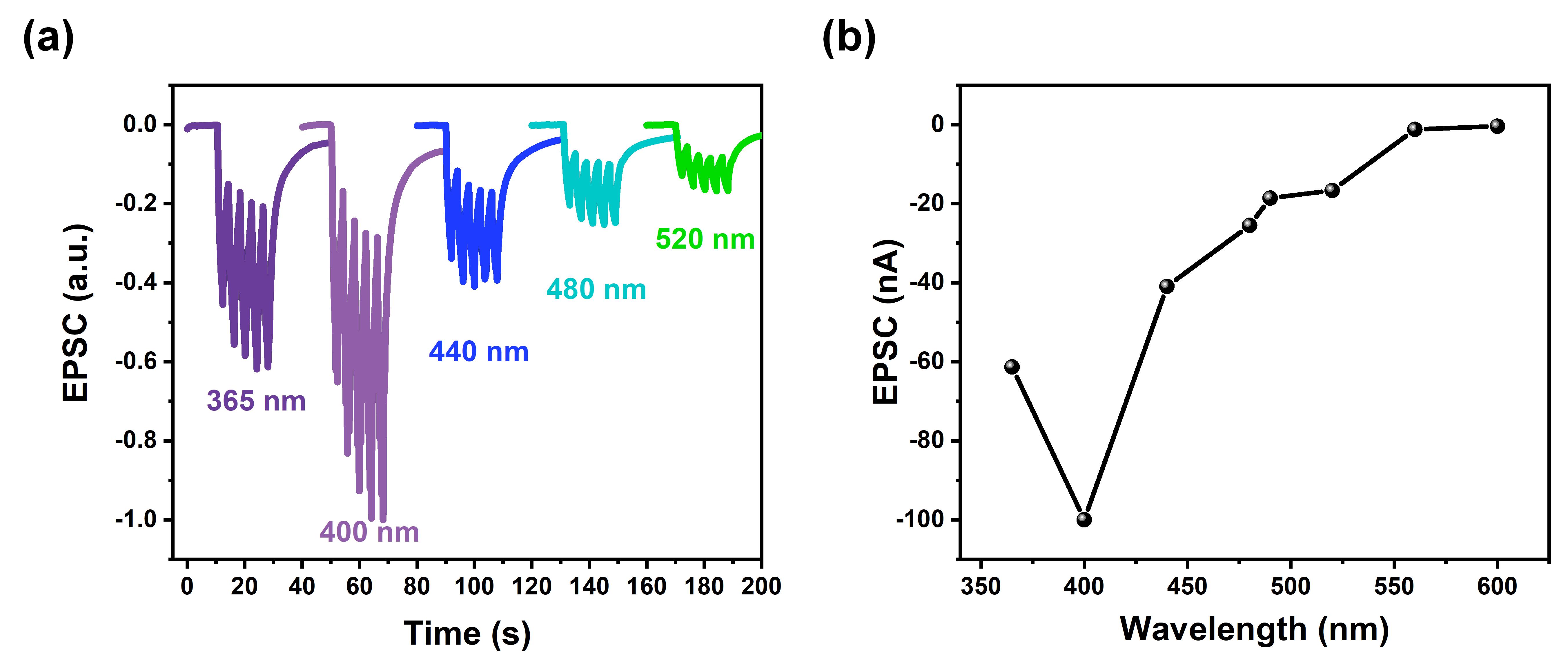


Figure S6. (a) Wavelength-dependent photocurrent of the photonic synapses based on CsPbBr_3_/solvent/CN when CN is illuminated (7.5 mW/cm^2^ and V_bias_ = 0 V). (b) The dependence of photocurrent on wavelength.


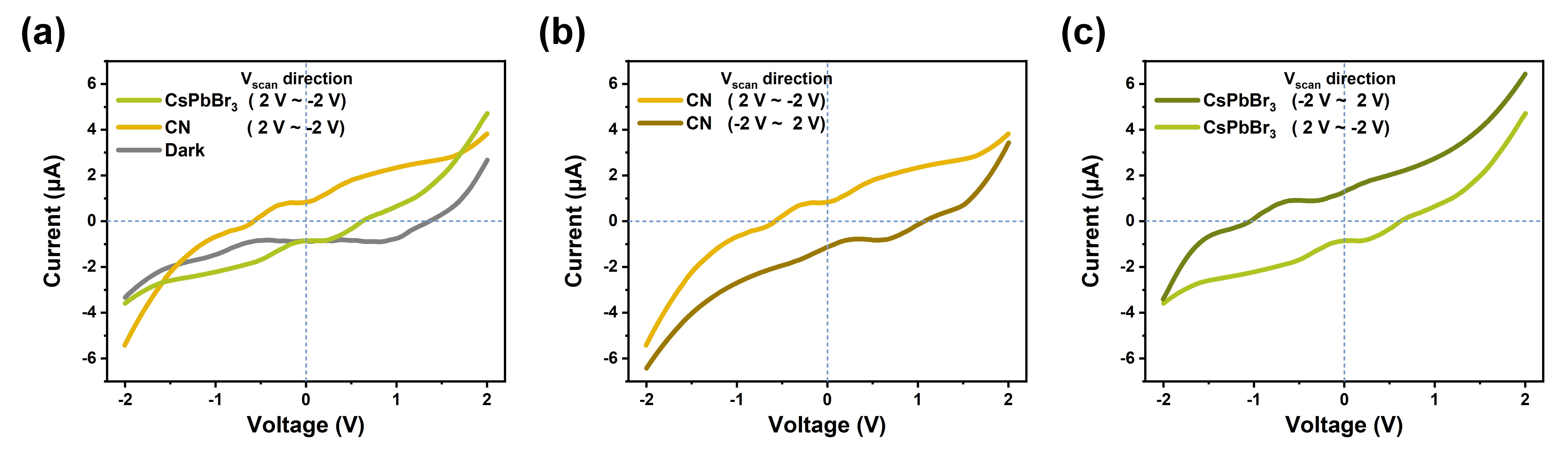


Figure S7. (a) I-V characteristic curves of the photonic synapses. (b) I-V characteristic curves of the photonic synapses under forward and reverse biases when CN (b) or CsPbBr_3_ are illuminated.

Figure S7a shows the I-V curves of the photonic synapses in both dark and light conditions. In the positive voltage range, the photo-response generated by illumination on CsPbBr_3_ is lower than that corresponding to CN. However, conversely, under the negative voltage range, the synapses exhibit a higher photocurrent when illuminated on the CsPbBr_3_ side, indicating that the gap in photocurrent cannot be solely attributed to the different photo-responsivities of CN and CsPbBr_3_. The magnitude of photocurrent strongly depends on the charge capture processes. Under the positive bias, photogenerated holes in CsPbBr_3_ QDs are captured by the solvent, thus the photocurrent corresponding to the irradiation on CsPbBr_3_ is smaller than that of CN, vice versa. Moreover, when the irradiation direction keeps unchanged, there is a significant hysteresis loop between the two I-V curves obtained from different scan directions (Figure S7b and c). Taking the illumination on CN side as an example, when the voltage is scanned from -2 V to 0 V, the synapses exhibit a negative photocurrent, corresponding to the photo-charging process of the synapse device. Due to the photovoltaic effect and discharging (negative current), the photocurrent keeps negative even the bias voltage is positive (0 to 1 V). The photocurrent does not reverse to positive until the V_bias_ approaches 1 V. On the other hand, when the bias voltage is scanned from 2 V to -2 V, the inflection point of positive and negative current values is -0.5 V, since the photo-charging process is negligible. Similar phenomenon is also observed when the illumination direction is changed to CsPbBr_3_ side.

Figure S8. Photocurrent of the synaptic device triggered by a UV pulse of 25 s duration and 25 mW/cm^2^ light intensity.


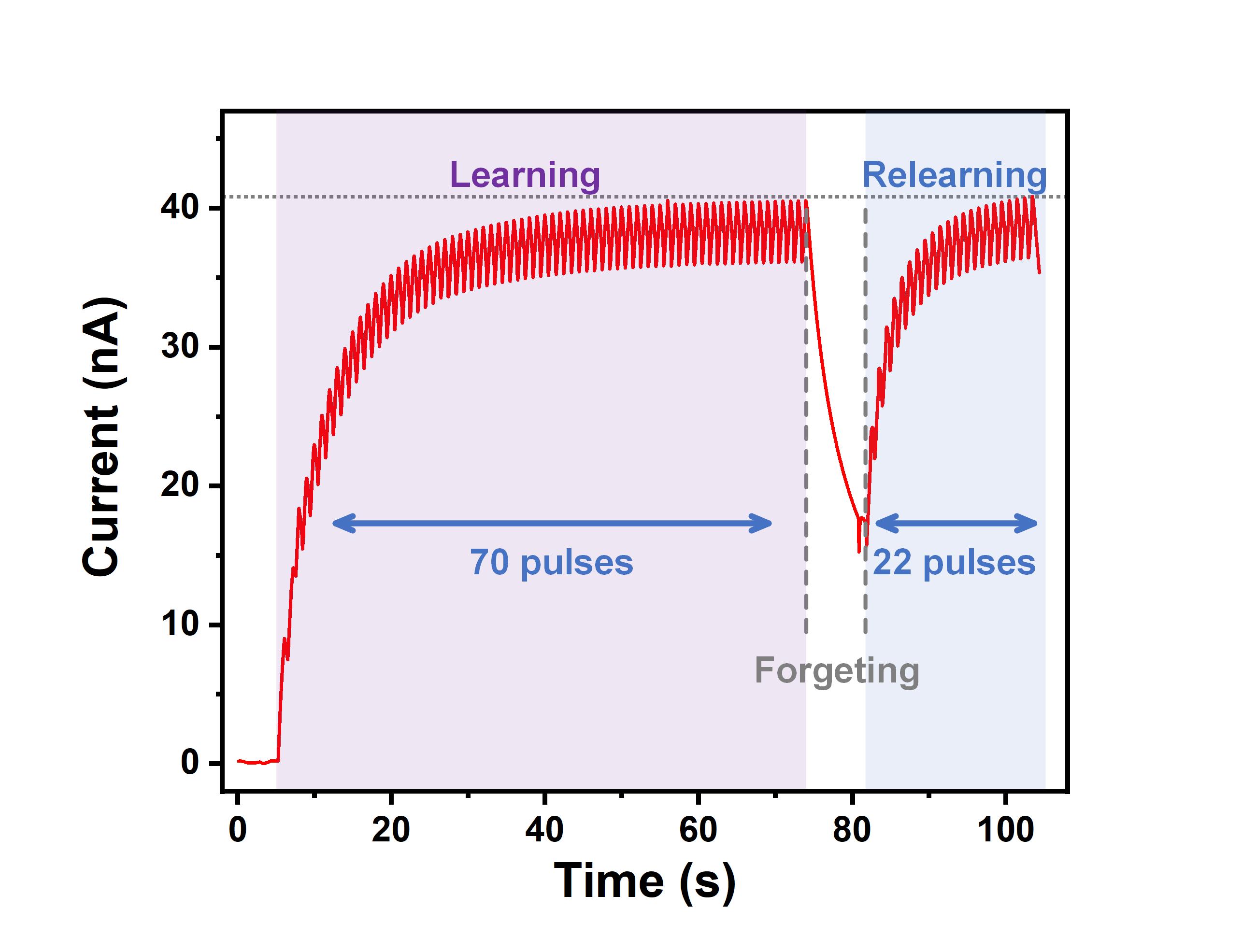


Figure S9. Learning, forgetting, and relearning processes of the photonic synapse under 365 nm light pulses (7.5 mW/cm^2^) with a pulse interval of 500 ms.

As shown in Figure S9, in the first learning process, the EPSC gradually increases to 42 nA when 70 consecutive light pulses are applied to the device. Then the EPSC decays to 70% of the maximum value, which is the forgetting process. For the case of relearning, only 22 light pulses are needed to restore the current to its previous state. This observation is consistent with the memory-forgetting curve of the human brain, wherein the time required for relearning is shorter than the first learning period.





Figure S10. (a) The EPSC triggered by 30 optical spikes at different spike frequencies. (b) The gain of EPSC (A_30_/A_1_) fitted with a sigmoidal-shaped function G_f_ = (a_1_-a_2_)/(1+(f/f_c_)^p^)+a_2_.

The incident light is a series of 30 spikes at different frequencies. As shown in Figure S10a, the EPSC amplitude increased dramatically as the input signal frequency increased from 0.5 to 10 Hz, suggesting the frequency dependence of the EPSC. Figure S10b shows the gain of the EPSC (G_f_), which is defined as the ratio of the thirtieth and the first optical pulses (A_30_/A_1_). The dependence of G_f_ on spike frequency can be well fitted with the sigmoidal function G_f_ = (a_1_-a_2_)/(1+(f/f_c_)^p^)+a_2_, which is a characteristic of the high-pass filtering observed in biological synapse. These results indicate that the photonic synapse may be used as high-pass filters for signal processing.


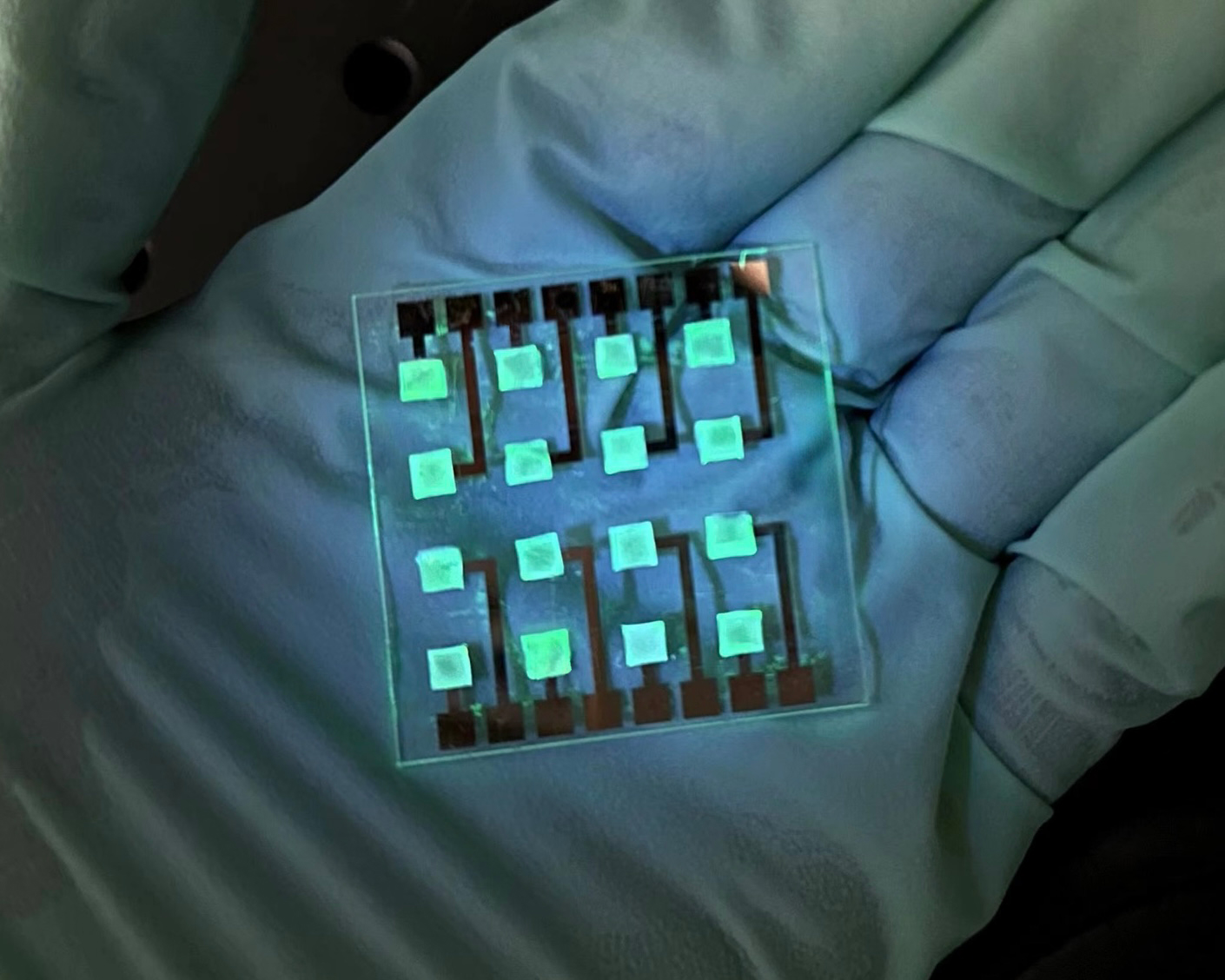


Figure S11. Photo of a real 4×4 device array.

**Supporting Note S1**

The memory ability of optoelectronic synapses makes it cannot rapidly switch different targets. Before initiating the next recognition task, waiting for complete photocurrent decay is time consuming. We noted that some recent studies have explored the use of inhibitory postsynaptic currents (IPSC) for optical erasing, which refers to the gradual declining of conductance by rising the number of optical pulses. For example, Li et al. developed a neuromorphic device based on the ZnO/PbS hybrid heterostructure that can mimic excitatory plasticity with the UV light, and IR light induces the inhibitory effect.^[S13]^ Hu et al. realized an all-optical controlled memristor based on IGZO, where the mem-conductance of the device can be reversibly modulated by altering the wavelength of the control light.^[S18]^ However, the IPSC in these studies is different to the optical erasing in our work. First, the EPSC and IPSC only occur under optical stimuli with different wavelengths, suggesting an additional optical excitation beam that different to the target signal is required (More details can be found in Table S2, Supporting Information). Second, the conductance gradually decreases under excitation of continuous optical pulses, implying it takes a relative long time to erase the postsynaptic current. Conversely, in our work, the optical writing and erasing respond to same optical stimuli. After completing a perception task or if there is a sudden need to switch target, the non-volatile photocurrent can be quickly reset to its initial state by reversing the stimulus direction. In addition, optical erasing also demonstrates a higher erasure efficiency compared to electrical erasing. Conventional electrical erasing necessitates the application of multiple reverse electrical pulses to restore the non-volatile current to its initial state (Table S2), typically requiring a considerable amount of time and power consumption. In contrast, optical erasing exhibits remarkable efficiency, enabling the rapid resetting of the non-volatile current with the application of a single light pulse.

Table S2. Comparison of power consumption, responsive wavelength range, PPF, retention time, and erasing function of photonic synapses based on different materials and structures.

| Three/two terminals | Materials | Power consumption* | λ | PPF (Δt) | Retention time | Erasing function | Ref |
| --- | --- | --- | --- | --- | --- | --- | --- |
| **Two** | **CsPbBr_3_/solvent/CN** | **Self-powered** | **365-520 nm** | **192% (0.5 s)** | **~300 s** | **Electrical erasing**  **Optical writing at 365 nm & erasing at 365 nm**  **<2 s** | **This work** |
| Three | CsPbBr_3_/PMMA | 1.4×10^-9^ J | 365-660 nm | 130% (1 s) | ~900 s | × | [S1] |
| Three | TIPS-pentacene/CsPbBr_3_ | 7.6×10^-10^ J | 450 nm | 143% (0.5 s) | N/R | × | [S2] |
| Three | C8-BTBT | 6.75×10^-16^ J | 300-500 nm | 145% (0.5 s) | ~300 s | × | [S3] |
| Three | Graphene/h-BN/CsPbBr_3_ | N/R | 520 nm | 196% (0.5 s) | N/R | Electrical | [S4] |
| Three | Cs_3_Bi_2_I_9_ | N/R | 405-635 nm | 120% (0.5 s) | ~120 s | Electrical | [S5] |
| Three | C_3_N_4_/PMMA/Pentacene | 1.81×10^-16^ J | 350-400 nm | 105% (0.5 s) | N/R | × | [S6] |
| Three | P3HT: PCBM | 3×10^-10^ J | 400-650 nm | 183% (0.5 s) | ~2 h | Electrical | [S7] |
| Two | P(VDF-TrFE)/Cs_2_AgBiBr_6_ | Self-powered | 445 nm | 137% (0.5 s) | ~50 s | × | [S8] |
| Two | CsPbBr_2_I/P3HT | Self-powered | 445-980 nm | 1.3% (0.5 s) | ~80 s | × | [S9] |
| Two | ZnO/P1/MoO_3_ | 2.85×10^-15^ J | 450-1850 nm | 125% (0.5 s) | ~60 s | × | [S10] |
| Two | SnO_2_/Al_2_O_3_/CsBi_3_I_10_ | Self-powered | 650 nm | 132% (0.5 s) | ~20 s | × | [S11] |
| Two | (BA)_2_PbI_4_ | 1.45×10^-16^ J | 400-600 nm | 125% (1 s) | ~100 s | Electrical | [S12] |
| Two | ZnO/PbS | 80×10^-12^ J | 365-980 nm | 45% | 100 s | Electrical set & reset | [S13] |
| Two | ZnO | N/R | N/R | N/R | 200 s | Optical set at 530 nm & reset at 650 nm | [S14] |
| Two | ZnO | N/R | 350-1000 nm | 124% | 300 s | Electrical set & reset | [S15] |
| Two | SnO_x_ | N/R | 254-533 nm | N/R | <5 s | Electrical set & reset | [S16] |
| Two | MoSSe/Al_2_O_3_/Li^+^/Al_2_O_3_ | N/R | 350-600 nm | 166% | 100 s | Electrical writing at 3 V & erasing at -3 V | [S17] |
| Two | OD-IGZO/OR-IGZO | N/R | 420-1000 nm | N/R | 200 s | Optical writing at visible & erasing at NIR  >20 s | [S18] |
| Two | MAPbBr_3_/ZnO | 1.8×10^-6^ J | 365-520 nm | 155% | 200 s | Optical writing at 365 nm & erasing at 520 nm via IPSC  100 s | [S19] |
| Two | Ag/TiO_2_ | N/R | 400-650 nm | 175% | 200 s | Optical writing at visible & erasing at UV via IPSC | [S20] |
| Two | PdSe_2_ | N/R | 473-1064 nm | 175%  (1064 nm)  180%  (473 nm) | 10 s | Optical writing at 1064 nm & erasing at 473 nm via IPSC | [S21] |
| Two | PMMA/Cs_2_AgBiBr_6_ | Self-powered | 350-1000 nm | N/R | N/R | Electrical set & Optical reset | [S22] |
| Two | TiO_2_/NiO | Self-powered | 375-512 nm | 144% | 20 s | Electrical set & reset | [S23] |

*: Power consumption for each synaptic event is calculated by *E* = *V*_bias_×*I*_light_×*T*_light_, where *V*_bias_ is the applied bias voltage, *I*_light_ is the peak value of EPSC, and *T*_light_ is the duration time of each optical spike.

Figure S12. Photocurrent of the photonic synapse right after fabrication and after six months of ambient storage. Pulse width=3 s, intensity=7.5 mW/cm^2^.

The device shows a good stability although the retention time of EPSC slightly reduced after storage as long as 6 months. The slight performance decay is primarily due to the relatively simplistic encapsulation technique employed, which has led to solvent evaporation. We would like to emphasize that in the future commercialization, the adoption of more stringent encapsulation methods is expected to further enhance its stability.

**Supporting Note S2**

We demonstrate that the photonic synapses can identify a target with the assistance of a very simple ANN. There are already many excellent algorithms, which have solved various complex tasks well. However, these neuromorphic algorithms still run on traditional von Neumann hardware, facing data shuttling bottlenecks and large energy consuming. As highlighted in numerous studies, the ultimate goal of developing photonic synapses is to replace the functionality of neural networks at the hardware level.^[S24-27]^ To highlight the pre-processing ability of our photonic synapses, we employed a very simple ANN in our simulation. The recognition accuracy of handwritten digit by the simple algorithm was only 11.4% (200 training epochs). With the pre-processing by our photonic synapses, the recognition accuracy of handwritten digit was significantly improved to 85% (~60 training epochs).


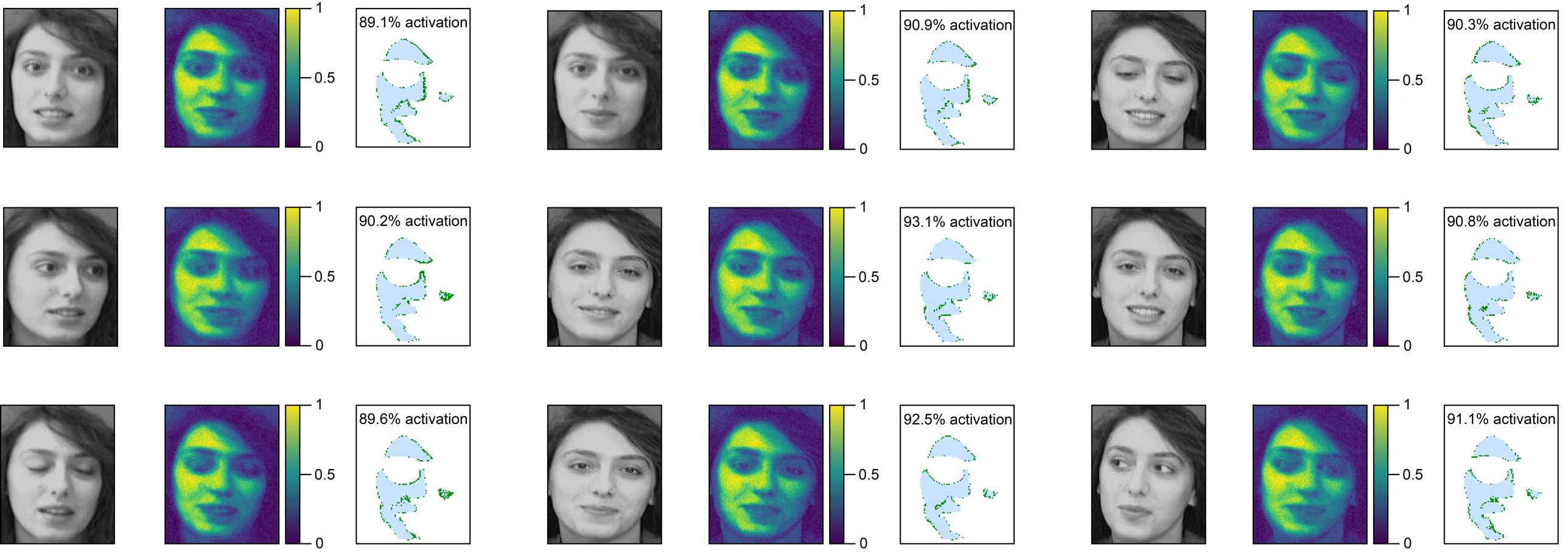


Figure S13. Face recognition tests using the target woman’s face with different angles and expressions.

# References

1. Y. Wang, Z. Lv, J. Chen, et al., Photonic Synapses Based on Inorganic Perovskite Quantum Dots for Neuromorphic Computing. *Adv. Mater.* 2018;30(38):1802883.
2. J. Liu, Z. Yang, Z. Gong, et al., Weak Light-Stimulated Synaptic Hybrid Phototransistors Based on Islandlike Perovskite Films Prepared by Spin Coating. *ACS Appl. Mater. Interfaces.* 2021;13(11):13362-13371.
3. Q. Li, T. Wang, Y. Fang, et al., Ultralow Power Wearable Organic Ferroelectric Device for Optoelectronic Neuromorphic Computing. *Nano Lett.* 2022;22(15):6435-6443.
4. C. Han, X. Han, J. Han, et al., Light‐Stimulated Synaptic Transistor with High PPF Feature for Artificial Visual Perception System Application. *Adv. Funct. Mater.* 2022;32(22):2113053.
5. Y. Li, J. Wang, Q. Yang and G. Shen. Flexible Artificial Optoelectronic Synapse based on Lead‐Free Metal Halide Nanocrystals for Neuromorphic Computing and Color Recognition. *Adv. Sci.* 2022;9(22):2202123.
6. H. L. Park, H. Kim, D. Lim, et al., Retina‐Inspired Carbon Nitride‐Based Photonic Synapses for Selective Detection of UV Light. *Adv. Mater.* 2020;32(11):1906899.
7. K. Chen, H. Hu, I. Song, et al., Organic optoelectronic synapse based on photon-modulated electrochemical doping. *Nat. Photonics.* 2023;17(7):629-637.
8. J. Lao, M. Yan, B. Tian, et al., Ultralow‐Power Machine Vision with Self‐Powered Sensor Reservoir. *Adv. Sci.* 2022;9(15):2106092.
9. X. Yang, Z. Xiong, Y. Chen, et al., A self-powered artificial retina perception system for image preprocessing based on photovoltaic devices and memristive arrays. *Nano Energy* 2020;78:105246.
10. S. Wang, H. Chen, T. Liu, et al., Retina‐Inspired Organic Photonic Synapses for Selective Detection of SWIR Light. *Angew. Chem. Int. Ed.* 2022;62(6):e202213733.
11. D. Hao, D. Yang, H. Liang, J. Huang and F. Shan. Lead-free perovskites-based photonic synaptic devices with zero electric energy consumption. *Sci. China Inf. Sci.* 2024;67(6):1-9.
12. Y. Wang, Y. Zha, C. Bao, et al., Monolithic 2D Perovskites Enabled Artificial Photonic Synapses for Neuromorphic Vision Sensors. *Adv. Mater.* 2024;36(18):2311524.
13. H. Li, X. Jiang, W. Ye, et al., Fully photon modulated heterostructure for neuromorphic computing. *Nano Energy* 2019;65:104000.
14. J. Yang, L. Hu, L. Shen, et al., Optically driven intelligent computing with ZnO memristor. *Fundamental Research* 2024;4(1):158-166.
15. T.-Y. Wang, J.-L. Meng, Q.-X. Li, et al., Reconfigurable optoelectronic memristor for in-sensor computing applications. *Nano Energy* 2021;89:106291.
16. S. P. Swathi, A. Makkaramkott and A. Subramanian. Tin Oxide Nanorod Array-Based Photonic Memristors with Multilevel Resistance States Driven by Optoelectronic Stimuli. *ACS Appl. Mater. Interfaces* 2023;15(12):15676-15690.
17. J. Meng, T. Wang, H. Zhu, et al., Integrated In-Sensor Computing Optoelectronic Device for Environment-Adaptable Artificial Retina Perception Application. *Nano Lett.* 2021;22(1):81-89.
18. L. Hu, J. Yang, J. Wang, et al., All‐Optically Controlled Memristor for Optoelectronic Neuromorphic Computing. *Adv. Funct. Mater.* 2020;31(4):2005582.
19. S. Ge, F. Huang, J. He, et al., Bidirectional Photoresponse in Perovskite‐ZnO Heterostructure for Fully Optical‐Controlled Artificial Synapse. *Adv. Opt. Mater.* 2022;10(11):2200409.
20. X. Shan, C. Zhao, X. Wang, et al., Plasmonic Optoelectronic Memristor Enabling Fully Light‐Modulated Synaptic Plasticity for Neuromorphic Vision. *Adv. Sci.* 2021;9(6):2104632.
21. J. Jiang, W. Xu, Z. Sun, et al., Wavelength‐Controlled Photoconductance Polarity Switching via Harnessing Defects in Doped PdSe_2_ for Artificial Synaptic Features. *Small* 2023;20(13):2306068.
22. H. Ye, Z. Liu, B. Sun, et al., Optoelectronic Resistive Memory Based on Lead‐Free Cs_2_AgBiBr_6_ Double Perovskite for Artificial Self‐Storage Visual Sensors. *Adv. Electron. Mater.* 2022;9(2):2200657.
23. C. Lu, J. Meng, T. Wang, et al., Fully Light Modulated Self-Powered Optoelectronic Memristor for Neuromorphic Computing. *IEEE Electron Device Lett.* 2023;44(10):1784-1787.
24. F. Zhou, Z. Zhou, J. Chen, et al., Optoelectronic resistive random access memory for neuromorphic vision sensors. *Nat. Nanotechnol.* 2019;14(8):776-782.
25. J. Jiang, W. Xiao, X. Li, et al., Hardware‐Level Image Recognition System Based on ZnO Photo‐Synapse Array with the Self‐Denoising Function. *Adv. Funct. Mater.* 2024;34(19):2313507.
26. Q. Liu, Q. Wei, H. Ren, et al., Circular polarization-resolved ultraviolet photonic artificial synapse based on chiral perovskite. *Nat. Commun.* 2023;14(1):7179.
27. S. Feng, J. Li, L. Feng, et al., Dual‐Mode Conversion of Photodetector and Neuromorphic Vision Sensor via Bias Voltage Regulation on a Single Device. *Adv. Mater.* 2023;35(49):2308090.
